# A new methodology for assessing health policy and systems research and analysis capacity in African universities

**DOI:** 10.1186/1478-4505-12-59

**Published:** 2014-10-08

**Authors:** Gillian Lê, Tolib Mirzoev, Marsha Orgill, Ermin Erasmus, Uta Lehmann, Stephen Okeyo, Jane Goudge, Stephen Maluka, Benjamin Uzochukwu, Moses Aikins, Don de Savigny, Goran Tomson, Lucy Gilson

**Affiliations:** Nuffield Centre for International Health and Development, Institute for Health Sciences, University of Leeds, Leeds, UK; Health Policy and Systems Programme/Health Economics Unit, School of Public Health and Family Medicine, University of Cape Town, Cape Town, South Africa; School of Public Health, Faculty of Community and Health, University of the Western Cape, Cape Town, South Africa; Tropical Institute of Community Health and Development, Faculty of Health Sciences, Great Lakes University of Kisumu, Kisumu, Kenya; Centre for Health Policy, School of Public Health, University of Witwatersrand, Johannesburg, South Africa; Institute of Development Studies, University of Dar Es Salaam, Dar Es Salaam, Tanzania; Health Policy Research Group and the Department of Health Administration and Management, College of Medicine, University of Nigeria Enugu-Campus, Enugu, Nigeria; Department of Health Policy, Planning and Management, School of Public Health, University of Ghana, Legon, Ghana; Health Systems Research and Dynamical Modelling Unit, Department of Public Health and Epidemiology, Swiss Tropical and Public Health Institute, Basel, Switzerland; Health Systems and Policy Research Group, Department of Public Health Sciences, Karolinska Institutet, Stockholm, Sweden; Medical Management Centre, Department of Learning, Informatics, Management and Ethics, Karolinska Institutet, Stockholm, Sweden; Department of Global Health and Development, Faculty of Public Health and Policy, London School of Hygiene and Tropical Medicine, London, UK

## Abstract

**Background:**

The importance of health policy and systems research and analysis (HPSR + A) has been increasingly recognised, but it is still unclear how most effectively to strengthen the capacity of the different organisations involved in this field. Universities are particularly crucial but the expansive literature on capacity development has little to offer the unique needs of HPSR + A activity within universities, and often overlooks the pivotal contribution of capacity assessments to capacity strengthening.

**Methods:**

The Consortium for Health Policy and Systems Analysis in Africa 2011–2015 designed and implemented a new framework for capacity assessment for HPSR + A within universities. The methodology is reported in detail.

**Results:**

Our reflections on developing and conducting the assessment generated four lessons for colleagues in the field. Notably, there are currently no published capacity assessment methodologies for HPSR + A that focus solely on universities – we report a first for the field to initiate the dialogue and exchange of experiences with others. Second, in HPSR + A, the unit of assessment can be a challenge, because HPSR + A groups within universities tend to overlap between academic departments and are embedded in different networks. Third, capacity assessment experience can itself be capacity strengthening, even when taking into account that doing such assessments require capacity.

**Conclusions:**

From our experience, we propose that future systematic assessments of HPSR + A capacity need to focus on both capacity assets and needs and assess capacity at individual, organisational, and systems levels, whilst taking into account the networked nature of HPSR + A activity. A genuine partnership process between evaluators and those participating in an assessment can improve the quality of assessment and uptake of results in capacity strengthening.

## Background

The importance of health policy and systems research and analysis (HPSR + A) has been increasingly recognised over the last decade [1–5] (for this paper, we use the acronym HPSR + A to include analysis (+A) in recognition that the field encompasses various forms of analysis undertaken by different actors). However, it is still unclear how to most effectively strengthen the capacity of HPSR + A [6]. Different types of organisations, such as universities, think tanks, Ministries of Health, NGOs, and health service delivery organisations, undertake HPSR + A. Of these, universities are particularly crucial to building the field because they not only produce knowledge but foster the next generation of policy-makers, health professionals, and researchers [7]. Nevertheless, debates within the expansive literature on capacity development appear to have little to offer the unique needs of HPSR + A activity implemented by individual researchers and groups within universities, especially where such groups have not been formalised into an academic unit such as a Centre or School. Furthermore, the pivotal contribution of capacity assessments to capacity strengthening processes is overlooked. This paper will help fill these gaps in the literature. Although we refer to universities, the assessment focused on HPSR + A groups which in some cases were defined academic units (e.g., a School) and in others cut across more than one academic unit (e.g., as was the case for the Nigerian partner). However, by including different levels within a university, a fair representation of the university as a whole in relation to HPSR + A was obtained.

Strengthening the capacity of HPSR + A groups within universities is a unique endeavour. HPSR + A has been defined as interdisciplinary research and analysis that aims to “understand how societies organise themselves to achieve collective health goals […] *how health systems respond and adapt to health policies, and how health policies can shape – and be shaped by – broader determinants of health*” ([5], p. 2). There are two key implications to consider from this. First, individual HPSR + A academics are usually scattered across traditionally organised academic units, which can create few opportunities for cross-departmental collaborative work. Second, since HPSR + A should be policy and practice relevant, conflicts arise between the scope of work of universities (including the criteria against which academics are assessed such as publications in academic journals) and the applied nature of the HPSR + A field that requires more emphasis on building relationships with policymakers and healthcare practitioners (often with less visible academic outputs). The stereotypical contrast is “*the researcher as independent scientist with a focus on the production of robust evidence with academic publication as the ultimate verification of achievement, versus the policy maker as a Mr/Ms ‘Fix it’ with a wide mandate to seek quick, workable solutions to current problems often based on compromise*” ([8, 9], p. 248). The stereotype is gross but has a purpose. It highlights the tensions experienced by university-based academics working in an applied field and seeking to be relevant to policy makers and practitioners but who must work to different resource constraints, timing, and performance outcomes (see also [10, 11]).

A plethora of frameworks exist for capacity assessments and strengthening of health and development organisations [12–16] and civil society research institutions [17], but none have been solely developed for the unique needs of HPSR + A within universities. A number of high quality assessments focusing on different aspects of the academic remit [18–21] have taken place, but there is no published assessments comparing HPSR + A capacity within and between different universities. Existing frameworks for generic capacity development emphasise the need to comprehensively focus on three levels of capacity – an individual working within an organisation, the organisation itself, and the wider system or environment in which an organisation operates. These levels are seen as interdependent: an individual has and requires knowledge and skills; organisations are the means by which individuals achieve (or not) collective goals; and the wider environment that constitutes the ‘rules of the game’ between organisations [18]. To comprehensively assess and strengthen capacity, all three levels should be addressed, either sequentially [16] or simultaneously [1, 13–18]. While capacity assessments increasingly focus on these three levels of capacity there is still little consideration on how the complex relations between the three levels of capacity can be strengthened. Existing frameworks also tend to focus on capacity needs with less consideration of potential assets. This is important because assets may exist but their application can often be constrained, for example, by a prohibiting environment.

The Consortium for Health Policy and Systems Analysis in Africa 2011–2015 (CHEPSAA) designed and implemented a novel capacity assessment for HPSR + A activity and groups within universities. The work was conducted during the first year of the project to support planning of project activities and to inform wider organizational development and networking in order to build the field of HPSR + A nationally, regionally, and internationally [7]. This paper reports on the methodology used and reflects on the lessons learned from designing and conducting that work, as well as setting out implications for other HPSR + A academics in any country considering the same.

A structured process was undertaken to draw out lessons from partner experience. Individual and group reflections were undertaken throughout the CHEPSAA assessment period. Partners’ reflected on the methodology in their context mapping and organisational/individual assessment reports, in emails, and at group sessions conducted at two annual consortium meetings in March 2012 and 2013, both of which were minuted. Authors also reviewed key project documents (methodology documents that guided assessments as well as comparative synthesis of partner reports) and, from these, identified three common themes. These were intended effects, unintended effects, and causes of confusion. Initial reflections were shared with all co-authors through several stages of reflection to confirm, challenge, and feedback into further refinement of the key lessons learnt in terms of building the capacity of the field.

The remainder of the paper is organised as follows. The description and process for developing the capacity assessment methodology is set out in next in detail. Thereafter, reflections are structured around four key lessons that are communicated to colleagues in the field and for those interested in capacity strengthening more generally.

### The CHEPSAA capacity assessment methodology

The participating partners in the CHEPSAA capacity assessment are identified in Table 1. The table summarises key features of these partners, at the time of the assessment. The institutional nature of the partners has been covered in detail elsewhere [7], therefore, in Table 1 below we emphasise their selected key features.

**Table 1:** **Key features of assessment partners**

Key features	Partners						
	Health Policy Research Group & the Department of Health Administration and Management, College of Medicine, University of Nigeria Enugu-Campus, Nigeria (HPRG-COMUNEC)	School of Public Health, University of Ghana, Ghana (SPH-UG)	Tropical Institute of Community Health and Development, Great Lakes University of Kisumu, Kenya (TICH-GLUK)	Institute of Development Studies, University of Dar Es Salaam, Tanzania (IDS-UDSM)	Health Policy and Systems Programme/Health Economics Unit, University of Cape Town (HPSP/HEU-UCT)	School of Public Health, University of the Western Cape (SOPH-UWC)	Centre for Health Policy, School of Public Health, University of Witwatersrand, South Africa (CHP-WITS)
Description/Reporting channel	Cross College research group within the Department; Department reports to College	School reports to University	Institute reports to University	Institute reports to University	Unit/Academic programme reports to School	School reports to University	Centre reports to School
Staffing	7 Health Policy and Systems Research and Analysis (HPSR + A) academics, 4 support staff	35 HPSR + A academics, 13 support staff	10 HPSR + A academics, 4 support staff	4 HPSR + A academics, 8 support staff	12 HPSR + A academics, 5 support staff	13 HPSR + A academics, 14 support staff	9 HPSR + A academics, 3 support staff
Research focus	Health policy and financing	Maternal health and human resources for health	Health policy and planning and health systems	Health policy	Health policy and systems, financing, economics and governance	Human resources, health policy and systems, information systems	Financing, human resources for health, universal coverage, health systems and policy

The assessment process included elements of self-assessment conducted by African partners, and external comparative synthesis conducted by a UK partner. Self-assessment was particularly attractive to the consortium because it was able to take advantage of existing research expertise within CHEPSAA. There was also a sense that the assessment process had potential to be a positive learning experience for all partners. The methodology development in the Consortium was led by one partner (University of Leeds), who initiated key documents, coordinated discussions at consortium meetings and at a distance through teleconferences and email exchange, and conducted comparative synthesis of partner findings.

Five principles underpinned the design of the methodology and conduct of the assessment.

**Figure 1:**
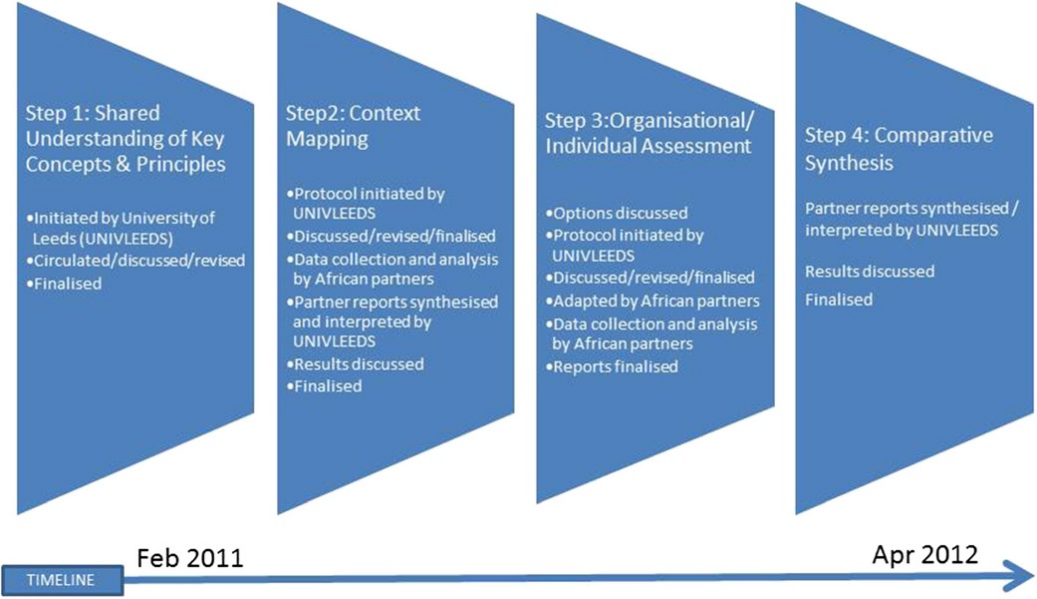
**Methodology overview.**

1. Three levels of capacity were incorporated in the assessment: individual, organisational and systems. This was done in order to conduct a comprehensive assessment, following consensus in the capacity development literature [1, 13–18].
2. An explicit focus on both assets and needs was adopted, to allow holistic assessment of the partners’ capacity rather than only focussing on current deficits. Partners recognised a number of pre-existing assets such as long experience with training programmes, links with national and regional health policy makers, and experience of networking across African institutions.
3. A semi-standardised approach was consciously chosen meaning that African partners agreed to return a minimum data set in their reports, to enable comparison of assets and needs at the consortium level. A fully standardised approach was not adopted because of the need for flexibility due to different organisational structures, existing capacity, and research foci.
4. A phased and incremental approach was taken as a practical way to ensure a) continuity of analysis alongside the data collection and b) spread of the workload of the assessment.
5. Last but not least, although the assessment was led by one partner, it was designed collaboratively between consortium partners. This was logical following the decision (at the outset) to partially conduct the assessment as a self-assessment and aimed to ensure ownership of both the assessment and subsequent capacity strengthening. Collaboration meant continuing review and conversations by email, teleconferences, and during annual consortium meetings. It also meant interim designs and results were extensively discussed and could be validated with partners. A collaborative approach also allowed time for individual and group reflections throughout.
  The methodology combined four broad steps over 14 months summarised in Figure 1 below.

The first step was to develop a shared understanding of the concept of capacity and capacity strengthening within the consortium. A concept note was circulated as a discussion document, which outlined key concepts, proposed the main principles, and suggested potential themes to assess HPSR + A capacity within African universities. The concept note was reviewed, discussed through at a teleconference involving all partners, and subsequently revised. During this process, a lack of clarity in understanding key concepts of the field became apparent. The Consortium Coordinator made a series of presentations and led discussions at the first annual consortium meeting to help develop a common sense of what constitutes HPSR + A.

**Figure 2:**
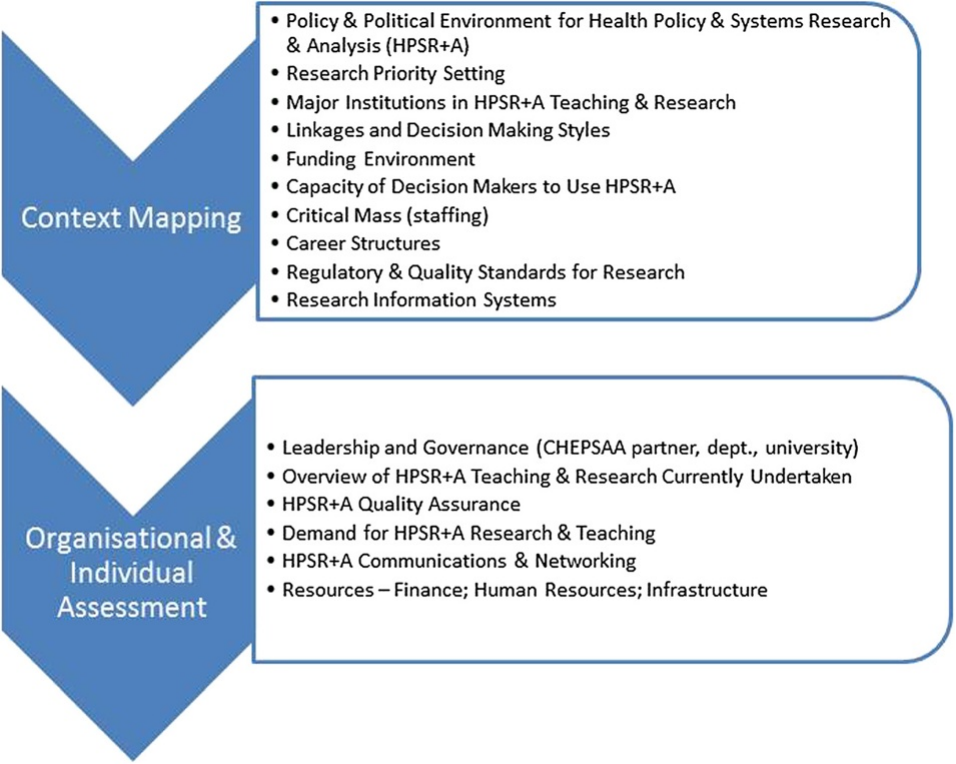
**Thematic areas of assessment.**

The second step was to map the contextual environment of HPSR + A for each CHEPSAA partner. A common guideline was developed, outlining the key areas for assessment, potential methods for data collection and analysis, and proposing a common structure of the report. The context mapping was anchored in a desk review but allowing for informal discussions with policy partners such as Ministries of Health, institutional partners, and reflection on personal experience by the HPSR + A academics. Partners developed individual reports (the three South African partners returned one combined report) against a minimum data requirement, which was subsequently synthesised using a framework approach based on assessment themes (Figure 2) to inform the next step in the assessment. The third step was a thematic organisational self-assessment incorporating individual assessment. The African partners returned individual reports that included relevant material from the context mapping. The fourth step was a comparative synthesis and subsequent discussion of assets and needs across the consortium. Again, a framework approach was used to analyse partner results and undertake a consortium-wide comparative synthesis. An assessment of current HPSR + A teaching materials used by partners was conducted separately.

The assessment focused on specific thematic areas (Figure 2). The thematic areas in the context mapping fed into development of the organisational and individual assessment themes. Greater detail on these themes is set out in the list below.

*Organisational and individual assessment: minimum information requirement*

1. **HPSR + A Leadership and Governance**
  1.1. Leader’s vision for HPSR + A research and teaching at three different levels – the CHEPSAA partner, the school/department in which the CHEPSAA partner is nested, and the university
  1.2. CHEPSAA partner and school/department organisational culture in terms of:
    1.2.1. Organisational structure
    1.2.2. How decision making takes place
    1.2.3. How communication of organisational vision, priorities, and activities occur
    1.2.4. Whether and how team building takes place
    1.2.5. Division of labour and definition of job roles
    1.2.6. Whether and how responsibilities and authority are allocated and, hence, how organisational succession planning occurs
    1.2.7. Lines of accountability for performance/non-performance
    1.2.8. Processes for giving rewards, bonuses, and promotions
  1.3. Organisational priority-setting for both HPSR + A research and teaching.
    (Characteristics could include: consideration of available funding and source vs. availability of qualified staff vs. areas of interest vs. funding/national priorities; consultative vs. hierarchical vs. ad hoc)
  1.4. Whether and how CHEPSAA partners have a financial strategy in place to support organisational priorities (1.3 above)
  1.5. Champions for both HPSR + A research and teaching – within in the school/department and/or the university
  1.6. Financial governance and regulations used within the CHEPSAA partner
  1.7. Central institutional support for, and CHEPSAA partner systematic mechanisms for, management of both HPSR + A research and teaching
  1.8. Future opportunities for strengthening both HPSR + A research and teaching – respondent views on how to build capacity in all its dimensions (both what would be desirable and what is actually feasible); deliberately open
2. **Overview of HPSR + A Research (only) Currently Undertaken by the CHEPSAA Partner**
  2.1. Extent of current HPSR + A research activities in terms of:
    2.1.1. Topic
    2.1.2. Total number of projects
    2.1.3. Total financial value across all projects
    2.1.4. Duration of projects
    2.1.5. Number of researchers per project
    2.1.6. Balance between HPSR + A research and other types of research
  2.2. Extent of current management only of research activities in terms of:
    2.2.1. Scope of activities
    2.2.2. Experience in this area and whether experience is relevant to the scope of work
    2.2.3. Challenges faced and support available in dealing with those challenges
  2.3. Future opportunities for strengthening the extent and availability of HPSR + A research and teaching – respondent views (both what would be desirable and what is actually feasible); deliberately open
3. **HPSR + A Research (only) Quality Assurance**
  3.1. Processes in use to ensure quality of research outputs; this topic could be approached through consideration of the following:
    3.1.1. Does the institution have written research guidelines (general and specific for HPSR)?
    3.1.2. Are there national research guidelines? If yes, what is the level of dissemination?
    3.1.3. What are the policy and legal issues relevant to HPSR + A?
  3.1.4. Which are the relevant regulatory institutions?
  3.1.5. What is the level of engagement in quality assurance?
  3.1.6. What is the status of strategic linkages of key stakeholder institutions?
  3.1.7. What strategic HPSR + A information/data is collected? And how regularly?
  3.2. Project monitoring and evaluation processes in use (incl. own or donor design)
  3.3. Ethical approval processes (incl. timing, complexity of application)
    3.3.1. What current approval procedures exist?
    3.3.2. Are there any specific requirements for HPSR + A research or need for the same, in contrast to other research
  3.4. Future opportunities for strengthening the quality of HPSR + A research processes and outputs – respondent views (both what would be desirable and what is actually feasible); deliberately open
4. **Demand for HPSR + A Research and Teaching**
  4.1. Policy/practitioner satisfaction with HPSR + A teaching and HPSR + A briefing notes/research syntheses when taken up (incl. recently expressed need of policy makers and managers in the field; whether and how policy maker/practitioners value HPSR + A research)
  4.2. What mechanisms exist for appraising and conveying HPSR + A needs of different stakeholders, consumers, and implementers
  4.3. Patterns of Overseas Development Assistance demand for CHEPSAA partner research outputs
  4.4. Patterns of government funded research undertaken by the CHEPSAA partner
  4.5. Student and staff satisfaction and concerns about a) current teaching/learning priorities, b) teaching style/approach, and c) whether teaching meets their competency needs
  4.6. Opportunities and mechanisms for student and staff exchange of ideas and experience
  4.7. Future opportunities for strengthening HPSR + A research and teaching demand, including any sense of where demand is currently unmet – respondent views (both what would be desirable and what is actually feasible); deliberately open
5. **HPSR + A Communications, Networking, and Getting Research Into Policy and Practice (GRIPP)**
  5.1. Socio-cultural communication norms within professional/academic formal and informal networks of the partner
  5.2. Perceived socio-cultural barriers and opportunities for developing research-policy maker-practitioner relationships
  5.3. Support for GRIPP in terms of:
    5.3.1. Frameworks within the CHEPSAA partner as well as region/country that enable GRIPP activities
    5.3.2. Identification and assessment of organisation’s role in national networks for both HPSR + A research and teaching
    5.3.3. Gatekeepers and channels incl. linkages between policy makers, practitioners, and research organisations; form and level of engagement with policy/practitioner makers
    5.3.4. Communication mechanisms in use including dissemination, feedback mechanisms, how HPSR + A outputs are packaged
  5.4. Description of case studies of networking and GRIPP that the institution has engaged in
    5.4.1. What are the lessons and best practices that can be recommended for scale up or replication?
  5.5. Perception of CHEPSAA partner by external funders and stakeholders – how do others see us?
  5.6. Coordination/harmonisation mechanisms between donors, research organisations, and government for HPSR + A research and teaching
  5.7. Future opportunities for strengthening future engagement between policy makers and practitioners for HPSR + A research and teaching – respondents views on how to build capacity in all its dimensions (both what would be desirable and what is actually feasible) and what improved future outcomes could be; deliberately open
6. **Resources – Finance**
  6.1. CHEPSAA partner funding patterns for both HPSR + A research and teaching in terms of:
    6.1.1. Total amount
    6.1.2. Sources
    6.1.3. Sustainability
    6.1.4. Relative balance between core vs. short term/donor funding for research
  6.2. Ability to identify, apply for, and obtain different funding streams that complement organisational priorities
  6.3. Effectiveness of internal information systems (incl. whether systems enable or undermine good management and why/how)
  6.4. Implementation of full cost recovery in external grant applications
  6.5. Future opportunities for strengthening financial systems to support HPSR + A research and teaching – respondent views (both what would be desirable and what is actually feasible); deliberately open
  **Resources – Human Resources**
  6.6. CHEPSAA partner existing academic staff:
    6.6.1. Age
    6.6.2. Gender
    6.6.3. Expertise (discipline/topic)
    6.6.4. HPSR + A specific research qualifications (e.g., undergraduate; postgraduate (Master/PhD), other)
    6.6.5. Experience of health systems research and teaching (incl. work experience in a previous relevant role/organization)
    6.6.6. Teaching qualifications (teaching diplomas, short courses with recognised accreditation)
    6.6.7. Teaching training undertaken but not accredited
  6.7. Academic staff turnover in terms of:
    6.7.1. Relative balance between academic staff engaged in HPSR + A on permanent/contract posts
    6.7.2. Minimum and maximum length of short contracts
    6.7.3. Number of contract renewals before termination
    6.7.4. Number of senior vs. junior vs. admin staff left/joined in the last 5 years
  6.8. Existing support staff in terms of:
    6.8.1. Number
    6.8.2. Age
    6.8.3. Gender
    6.8.4. Expertise
    6.8.5. Years of experience in project administration/finance/communication
  6.9. All staff: in the last 5 years, awareness and uptake of any staff development/support activities
  6.10. All staff: felt need for any of the following:
    6.10.1. HPSR + A technical skills
    6.10.2. HPSR + A research and writing
    6.10.3. HPSR + A teaching
    6.10.4. Human resources skills
    6.10.5. Management and administration
    6.10.6. Financial strategy
  6.11. Future opportunities for strengthening HPSR + A research and teaching by building on, retaining and taking advantage of, current human resources assets – respondent views (both what would be desirable and what is actually feasible); deliberately open
  **Resources – Infrastructure**
  6.12. Appropriate office space available for academic and support staff in research and teaching (incl. meeting/classroom space, etc.)
  6.13. Research resources available (incl. IT hardware and software – quality and availability of internet connection; IT staff support with training/use; teleconferencing facilities; paper and electronic libraries access incl. subscription to journals)
  6.14. Teaching resources available (incl. equipment)
  6.15. Reliability of basic services supply (e.g., electricity) and availability of alternative sources (e.g., generator)
  6.16. Key infrastructural challenges and how they influence current research and teaching as well as how they are (or not) addressed – respondent views (both what would be desirable and what is actually feasible); deliberately open

As part of the commitment to a semi-standardised approach, a menu of methods was proposed to allow CHEPSAA partners to select those most appropriate for their setting with different respondent groups (Table 2). These were: i) document review particularly internal documents; ii) in-depth interviews with key respondents; iii) focus group discussions; iv) a staff survey template; and v) participatory stakeholder workshop.

**Table 2:** **Respondents**

	Respondents	Examples
**University – Internal**	CHEPSAA Health Policy and Systems Research and Analysis (HPSR + A) team	Staff on HPSR + A research and teaching projects
	Other university employed staff with whom the CHEPSAA partner has necessary working relationships	Colleagues from cross cutting departments (e.g., finance, human resources, and research quality assurance
	Leaders	CHEPSAA partner/school/department/university
**Stakeholders – External**	Users of HPSR + A research and teaching	Current and former students/teachers from other HPSR + A research and teaching institutions
	Core and overseas key funders for both research and teaching	Donor staff at senior and project level/senior and midlevel bureaucrats in core grant administration and prioritisation
	Major institutions in HPSR + A research and teaching identified in the context mapping	Politicians/ministry department leaders at national, regional, or local bureaucrats responsible for policy drafting
	Provincial Ministry departments and research centres, as well as Research Committees	
	Peer organisations	Formalised networks/professional associations

Partners used varying combinations of these methods (Table 3), although the participatory stakeholder workshop was not used.

**Table 3:** **CHEPSAA assessment methods by partner**

Partner	Methods used
Health Policy Research Group and the Department of Health Administration and Management, College of Medicine, University of Nigeria Enugu-Campus, Nigeria (HPRG-COMUNEC)	▪ Document review (e.g., National code for research ethics)
	▪ In depth interviews x 27
	▪ Focus group discussions x 4
	▪ Staff survey (College) of 121 respondents
School of Public Health, University of Ghana, Ghana (SPH-UG)	▪ Document review (e.g., annual reports from research and development division of the Ghana Health Service)
	▪ In depth interview x 1
	▪ Staff survey of 57 respondents
	▪ Focus group discussions (using NetMap tool) x 3
Tropical Institute of Community Health and Development, Great Lakes University of Kisumu, Kenya (TICH-GLUK)	▪ Document review (e.g., programme and research reports, case study reports)
	▪ In-depth interviews x 78
	▪ Focus group discussions x 9
	▪ Staff survey of 7 respondents
	▪ Student survey of 98 respondents
Institute of Development Studies, University of Dar Es Salaam, Tanzania (IDS-UDSM)	▪ Document review (e.g., analysis of key policy documents)
	▪ In depth interviews x 25
	▪ Staff and former student survey of 31 respondents
Health Policy and Systems Programme/Health Economics Unit, University of Cape Town, South Africa (HPSP/HEU-UCT)	▪ Document review (e.g., UCT guidelines on assessment of staff performance)
	▪ In-depth interviews x 13
	▪ Focus group discussions x 2
	▪ Staff survey of 13 respondents
School of Public Health, University of the Western Cape, South Africa (SOPH-UWC)	▪ Document review (e.g., SOPH annual reports, project management guidelines)
	▪ In-depth interviews x 9
	▪ Focus group discussion x 1 (which included a mini staff survey of 9 respondents)
Centre for Health Policy, School of Public Health, University of Witwatersrand, South Africa (CHP-WITS)	▪ Document review (e.g., context mapping report, CHP annual reports)
	▪ In depth interviews x 11
	▪ Staff survey of 7 respondents

There are limitations in the assessment methodology that has been presented here. The value of including elements of self-assessment has been discussed but there is also potential that important issues may have been missed because of the predominantly ‘insider’ perspective in collecting and analysing the results within each organisation. While the assessment was not solely self-assessment, the participating external partner was a partner of the research consortium and therefore not strictly an outsider. Limitations of self-reporting can be poor recall. Social desirability bias is also possible, meaning under reporting of what the reporter perceives to be negative and over reporting of what is perceived by a reporter to be positive. There is also scope for over reporting of deficits to make the case for future additional resourcing of HPSR + A units. The CHEPSAA methodology sought to take account of these limitations. There was active engagement of all partners in developing and applying methodology in capacity assessments. In addition, the semi-standardised methodology meant that common thematic areas (Figure 2) were accompanied by a list of required information agreed by all Consortium partners (see *Organisational and individual assessment: minimum information requirement* above). Therefore, a combined weight of experience from different disciplines, institutions, and individuals was brought to the assessment. The results of the capacity assessment have been published elsewhere [7] and are not reprised here.

### Reflections

The approach taken was felt by partners to be overwhelmingly positive (discussed later) suggesting that it can be considered appropriate and relevant to the contexts and concerns of HPSR + A units. From sustained reflection on the development and conduct of the CHEPSAA assessment methodology described above, four lessons can be communicated. First, the CHEPSAA methodology is new and unique to the field of HPSR + A. Second, in HPSR + A the unit of assessment can be a challenge, because HPSR + A groups within universities tend to overlap between academic departments and are embedded in different external networks. Third, a capacity assessment is not only a planning tool but is also capacity strengthening of itself. Last, based on the CHEPSAA experience, a number of methodological issues for consideration in future capacity assessments can be proposed. Each lesson is discussed in turn below.

### A new capacity assessment methodology for HPSR + A

Although universities play a vital role in HPSR + A, there are currently no capacity assessment methodologies for HPSR + A groups within universities. Assessment of administrative, decision making, and political capacity of health ministries [19, 20] and research and analytical capacities of independent research institutes [21], has been undertaken. Capacity assessments that have been conducted within universities have covered specific training courses, such as public health [22], post-graduate epidemiological research [23], and health services and systems training [24], or a transdisciplinary aspect of the academic remit such as knowledge translation [25] or running doctoral programmes [26]. None of these have assessed the field of HPSR + A solely within a university and none has done so comprehensively across three levels of capacity. The CHEPSAA assessment methodology is therefore new, unique, and implemented across seven institutions. It contains insights that could be of potential use to HPSR + A units in other universities around the world who can adopt or evolve the methodology for their local contexts. It can also be useful to HPSR + A units in other institutions such as Ministries of Health or civil society organisations. However, their specific scope of work will need to be reflected in the assessment themes (*c.f.* Figure 2). For instance, HPSR + A Quality Assurance may not be a priority for a Ministry of Health but other themes such as Leadership and Governance, Communications and Networking, and Resources would continue to be relevant.

### Capacity assessments take account of the networked nature of HPSR + A

Methodologically, it was often difficult to pin down ‘who’ was being assessed. The unit of assessment was more fluid than we were led to expect from accounts of other capacity assessments. In those accounts, the individuals assessed are seen as nested discretely within autonomous organisations that have complete control over its available resources. This was not the case for HPSR + A groups within universities.

The organisation in the CHEPSAA assessment was initially taken to be the contracted partner, which is an administrative unit within a university. Each unit has a different structural relationship to the university in which it is housed (Table 1). The Health Policy Research Group, College of Medicine, University of Nigeria Enugu-Campus (HPRG-COMMUNEC), for instance, is a cross college research group that sits within a department of the college but whose membership and remit is not constrained by the department. The Health Policy and Systems Programme/Health Economics Unit, University of Cape Town (HPSP/HEU-UCT) was, at the time of the assessment, a teaching and research programme closely allied with the Health Economics Unit within the School of Public Health and Family Medicine. The largest partner was the School of Public Health, University of the Western Cape (SOPH-UWC) where the whole school had an overt HPSR + A remit, and hence the School itself was assessed. The organisational assessment required partners to inquire into the assets and needs of different capacity themes such as organisational leadership, governance, and resourcing (Figure 1). However, this led to confusion. University financial governance structures, for instance, were not unique to those working in HPSR + A; provision of teaching and research aids were not solely generated or used by HPSR + A units (whether a research group, a department or a school). The School of Public Health, University of Ghana (SPH-UG) assessed all research individuals working in the School but noted that “*HPS(R) + A type research is carried on in many schools and departments…* [within] *University of Ghana* [this not only includes the School of Public Health but also] *political science department, sociology department …* [it is] *difficult to be clear as to the exact extent given the absence of any coordinating mechanism and … that much of the output may remain in the grey literature*” ([27], p. 20). The assessment captured some of the individuals working either full time or part time in HPSR + A since it was assumed that individuals participating in the assessment should be those nested within the organisational unit. However, this meant that individuals working on HPSR + A outside of the CHEPSAA partner were not included in the assessment. In other words, the CHEPSAA partner was not a freestanding organisational unit with a clear boundary.

Partners also included external partners as part of their assessment but this meant including the capacity assets and needs of these stakeholders. For instance, HPRG-COMMUNEC also included capacity assets and needs of policy makers; the Tropical Institute of Community Health and Development, Great Lakes University of Kisumu (TICH-GLUK) worked with non-academic organizations that are active in health policy as well as policy implementers such as the District Health Management teams. The inclusion of external partners was essential to the CHEPSAA assessment given that HPSR + A is an applied field.

However, when seeking to apply the generic capacity development levels used in existing frameworks, the boundary of the organisational unit in the CHEPSAA assessment became fuzzy. In retrospect, partners solved the confusion by approaching the unit flexibly, paying attention to the existence and quality of internal relationships between individuals and departments within the university, and externally with policy makers and practitioners. Future HPSR + A capacity assessments within universities need to be aware of the networked nature of the field and that this may change over time. At present, the field is embedded within different units and linkages internal to a university. This may change as the field grows when, for instance, there may be more Schools such as SOPH-UWC wholly focused on HPSR + A. However, the field is also heavily committed to strong relationships with external stakeholders (see earlier definition) and these should only endure and strengthen as the field matures.

Each HPSR + A academic unit in the CHEPSAA assessment has a very different institutional nature hence there can be no standard approach to charting a network. However, one possible way of doing so would be to revisit the tasks undertaken by HPSR + A staff and a HPSR + A unit to understand the relative balance between responsibility for tasks and governance/oversight of those tasks. For example, staff within a centre may undertake teaching across programs but employment of such staff is at the discretion of faculty. A future assessment would then explore how to strengthen those networks in the interests of building the field.

### Capacity assessments can themselves be capacity strengthening

A capacity assessment is usually seen as a planning tool that can help organisations plan, strategize, and make decisions on future capacity strengthening activities [2, 12, 17]. The CHEPSAA assessment was used in this way. For instance, in CHEPSAA, the information gathered on the theme of Demand for Research and Teaching (Figure 2) and from staff surveys in which academic staff expressed a desire to teach more which, together with the concurrent review of HPSR + A course material, ultimately led to the development of two open access modules on complex health systems and health policy and systems research.

However, a capacity assessment can itself strengthen capacity. CHEPSAA partners found the inclusion of self-assessment to have unintended positive consequences. SOPH-UG, for instance, used it as an opportunity to synthesise “*bits and pieces*” of HPSR + A information scattered in annual reports, job descriptions, grant proposals, meeting minutes, academic and grey reports, and mass media sources into a coherent sense of what HPSR + A meant for the School. For SOPH-UWC, it helped them question whether they were taking sufficient advantage of existing internal assets within UWC and to think about how to prioritise future activities.

The assessment also had a powerful awareness raising effect that has not yet been reported in other assessments. There were two aspects to this. First, partners and respondents (Table 2) became more aware of their different conceptual understandings of HPSR + A and this led to efforts to develop a common understanding of the field. Given such differences, the researchers independently developed an information sheet to provide a coherent definition of HPSR + A and to act as a starting point for discussion about the field. Second, by working from this common base, respondents could more easily identify existing assets and recognise future opportunities, as well as future applications of these assets. For example, HPSP/HEU-UCT responded to ideas developed during assessment interviews to establish a journal club between interested academics and government managers in Cape Town to allow continued engagement and development of understandings around the terrain of HPSR + A. TICH-GLUK incorporated the assessment process into an existing mentorship programme by ensuring the assessment team were in mentor-mentee-peer support relationships. Following the assessment, the Centre for Health Policy, School of Public Health, University of Witwatersrand (CHP-WITS) developed a business case for HPSR + A teaching to present to university leadership and senior management and ultimately secured two centrally funded posts for early career academics in recognition of the unit’s contribution to teaching. Many respondents at the Institute of Development Studies, University of Dar Es Salaam (IDS-USDM) assessment expressed the need to establish a national network of HPSR + A academics and the first steps have now been taken to establishing a network of six universities teaching in the field. In other words, a significant unintended positive consequence of the capacity assessment process was that it produced outcomes associated with capacity strengthening interventions. This is consistent with Horton’s assertion that “*every evaluation* [assessment] *of a capacity development effort should itself contribute to… capacity… and ultimately to the organization’s performance*” [28].

It is acknowledged here that doing capacity assessments requires a certain level of capacity. The methodology described earlier was built through collaboration, but collaboration has a cost. Staff time, IT infrastructure and software, and a travel budget were required for collaboration by all participating partners. Resources were required to undertake data collection and analysis – for instance, ethical approvals were obtained for fieldwork; significant time was spent setting up appointments for interview and focus groups with policy makers, many of whom had competing schedules; TICH-GLUK notably trained staff to act as survey enumerators for an element of staff skills assessment. Not least, resources were also necessary to synthesise data, write reports, and conduct comparative analyses. This does not negate the capacity strengthening effect of assessments, but does mean that assessments should be designed to make the best use of existing capacity, rather than overstretch it.

### Implications for future capacity assessments

The capacity assessment methodology reported here was developed and implemented across a diverse range of African universities. Even though our focus was specifically on African universities, we believe our results are also largely applicable to universities in other lower middle income countries with yet to fully establish HPSR + A within its institutions, because the nature of HPSR + A work is likely to be similar and will include different combinations of teaching, research including its communication, consultancies and advisory functions, and networking activities. However, one possible significant difference which may affect the adoption of our methodology relates to resource environments within different contexts. For HPSR + A units in other African universities and other continents wishing to conduct such an assessment, a number of methodological considerations are proposed.

First, consider including deliberate elements of self-assessment because it can be empowering for staff to engage directly with colleagues and leaders that can significantly influence the field. The potential limitations of self-assessment can be mitigated through standardising a number of components, ensuring a common understanding of key terms and methods, and continual engagement between partners in development and implementation of assessment methodology. Second, consider assets as well as needs because it is more likely to identify resources that teams may not be aware of or taking sufficient advantage of. Third, address the capacity potential of three levels – individual, organisational, wider environment – since doing so will help plan more comprehensive capacity strengthening activities. However, be aware that HPSR + A activities are embedded in different networks between and within these level – a more networked approach to assessing capacity has been proposed in an earlier section. Fourth, take a phased and incremental approach because it is both practical in terms of time and resources available and can give time to assessment teams to reflect on what they are learning in the process since it allows iterative data collection and analysis. Fifth, use methods and tools that assessment teams are familiar with because it makes best use of existing resources. Using unfamiliar methods and tools in an assessment incorporating self-assessment means that capacity development will be necessary before the assessment can begin. It is not always necessary to develop new methodological skills during a capacity assessment.

Finally, seek and fully cost genuine collaboration from the beginning because genuine collaboration means ownership of process and outcome but does require significant time, finance, and other resources to be invested (also noted in [21, 29]). When collaborating across large consortia, such investment will pay off in clear leadership at consortium and partner level and a clear methodological framework. Even where assessments do not include elements of self-assessment, genuine partnership between external assessors and those being evaluated can deepen external understanding of the everyday concerns of those participating in an assessment, and vice versa, and help those being evaluated to better understand the value of assessment for capacity strengthening.

## Conclusions

This paper has reported a new capacity assessment methodology which was used by seven universities in five African countries for HPSR + A. Sustained reflection on the development and conduct of the assessment identified key lessons for HPSR + A colleagues working in this nascent field and still fighting for profile and resources. First, the methodology for capacity assessments of universities in relation to HPSR + A reported here is a first of its kind and can contribute to building the field by providing guidelines for future capacity assessments. Second, since HPSR + A in universities is likely to always have fluid boundaries, a more networked approach to capacity assessments and capacity strengthening is appropriate. Third, capacity assessments are themselves capacity strengthening, even though initial capacity is required to conduct these assessments. Last, based on the CHEPSAA experience, it is proposed that future systematic capacity assessments focus on both capacity assets and needs; assess capacity at individual, organisational, and systems levels; consider self-assessments using familiar data collection and analysis methods; and conduct assessments in a phased and incremental way.

## Abbreviations

CHEPSAA: Consortium for Health Policy and Systems Analysis in Africa 2011–2015
CHP-WITS: Centre for Health Policy, School of Public Health, University of Witwatersrand, South Africa
HPRG-COMUNEC: Health Policy Research Group and the Department of Health Administration and Management, College of Medicine, University of Nigeria Enugu-Campus, Nigeria
HPSP/HEU-UCT: Health Policy and Systems Programme/Health Economics Unit, University of Cape Town, South Africa
HPSR + A: Health policy and systems research and analysis
IDS-UDSM: Institute of Development Studies, University of Dar Es Salaam, Tanzania
SOPH-UWC: School of Public Health, University of the Western Cape, South Africa
SPH-UG: School of Public Health, University of Ghana, Ghana
TICH-GLUK: Tropical Institute of Community Health and Development, Great Lakes University of Kisumu, Kenya.
